# Longitudinal Assessment of the Canine Fecal Microbiota in Response to Dietary Hempseed By-Product and Oil: A 90-Day Nutritional Intervention Study

**DOI:** 10.3390/vetsci13060534

**Published:** 2026-05-29

**Authors:** Jutamat Klinsoda, Sasithorn Limsuwan, Witchayaporn Sornard, Pattarawadee Thamsatit, Natthasit Tansakul

**Affiliations:** 1 Institute of Food Research and Product Development, Kasetsart University, Bangkok 10900, Thailand; ifrjmk@ku.ac.th (J.K.); sasithorn.limsu@ku.th (S.L.); 2 Special Research Incubator Unit for Cannabis-Hemp and Phytochemicals in Veterinary Medicine, Faculty of Veterinary Medicine, Kasetsart University, Bangkok 10900, Thailand; 3 Graduate Program in Animal Health and Biomedical Sciences, Faculty of Veterinary Medicine, Kasetsart University, Bangkok 10900, Thailand; witchayaporn.sor@ku.th; 4 Kasetsart Veterinary Teaching Hospital, Faculty of Veterinary Medicine, Kasetsart University, Bangkok 10900, Thailand; gracepatt789@gmail.com; 5 Department of Pharmacology, Faculty of Veterinary Medicine, Kasetsart University, Bangkok 10900, Thailand

**Keywords:** canine microbiome, hempseed by-product, hempseed oil, gut microbiota, functional ingredients

## Abstract

Industrial hemp (*Cannabis sativa* L.) derivatives are emerging as functional ingredients in companion animal nutrition; however, their long-term effects on the canine gut microbiome remain unclear. This exploratory study investigated the impact of dietary supplementation with two hempseed-based formulations (fiber-rich by-product vs. fat-rich oil coating) on the fecal microbiota of healthy adult dogs over 90 days. Twenty-four dogs were randomly assigned to control, hempseed by-product (11% inclusion), or hempseed oil (2% coating) diet groups. Fecal samples collected on days 0, 30, and 90 were analyzed using 16S rRNA gene sequencing to assess microbial composition and diversity. The hempseed oil group showed a distinguishable increase in species richness. Both hempseed-based diets were associated with compositional shifts in formulation-specific ways: the oil coating with a higher relative abundance of *Lactobacillaceae* (notably *Ligilactobacillus*), and the by-product with a higher relative abundance of Actinobacteriota (particularly *Collinsella*). Both treatments preserved several microbiota genera. Beta diversity analysis revealed significant temporal restructuring, with convergence toward a stabilized ecosystem by day 90. These findings demonstrate that hempseed fractions modulate the canine microbiome in a formulation-specific manner without disrupting ecological stability, supporting beneficial health effects in canine nutrition.

## Simple Summary

This study explored the effects of two different hempseed-based ingredients, hempseed oil and a hempseed by-product, on the gut health of healthy adult dogs over a 90-day period. All dogs, including the controls, showed a natural increase in bacterial diversity over time. However, dogs fed hempseed oil showed a distinctly larger increase in bacterial species richness. The diet containing the hempseed by-product, on the other hand, specifically promoted the growth of certain beneficial bacteria. Importantly, neither treatment disrupted the microbial community essential for gut stability. These results suggest that hempseed derivatives altered microbial composition by promoting certain beneficial bacteria, supporting canine gut health in a formulation-dependent manner, and provide evidence for their safe inclusion as functional ingredients in dog food.

## 1. Introduction

The gastrointestinal tract of mammals hosts a complex and dynamic community of microorganisms, collectively referred to as the gut microbiota, which functions as a critical immune and metabolic organ [1,2]. In healthy dogs, this microbial community exists in a state of eubiosis, characterized by a diverse and balanced composition that produces beneficial metabolites, such as short-chain fatty acids (SCFAs), which support intestinal barrier integrity, immune regulation, and metabolic homeostasis [2,3]. While the canine microbiome is influenced by intrinsic factors such as age and breed, diet remains the most significant extrinsic modulator of its taxonomic structure and functional output [4,5].

Industrial hemp (*Cannabis sativa* L.), defined as varieties containing <0.3% Δ^9^-tetrahydrocannabinol (Δ^9^-THC), has gained considerable attention as a sustainable and nutritionally rich component of animal feed [6]. Hempseeds are particularly versatile, offering high-quality proteins, such as edestin and albumin, alongside a unique lipid profile rich in polyunsaturated fatty acids (PUFAs), which are beneficial for animals [7,8,9]. Specifically, hempseed oil contains high concentrations of essential fatty acids, including linoleic acid (C18:2 n-6) and alpha-linolenic acid (C18:3 n-3), typically in a 3:1 ratio, which is considered optimal for supporting canine health and anti-inflammatory pathways [10,11]. Additionally, the processing of hempseed yields valuable by-products, which provide residual lipids and high concentrations of insoluble fiber [6,8,10].

Despite the established nutritional profile of hemp-derived products, scientific research on their impact on the canine gut microbiome remains limited and is predominantly cross-sectional [10,12]. Longitudinal studies are essential for understanding how these ingredients influence the temporal dynamics, ecological stability, and maturation of the gut environment [5,13]. While some preliminary data suggest that hemp supplementation can increase beneficial taxa, such as *Lactobacillus* and *Bifidobacterium* [14,15], the long-term ecological consequences of substituting different hempseed fractions (oil versus by-products) in canine diets have not been fully elucidated [16,17].

To address this gap, the present study utilized 16S rRNA gene sequencing to perform an exploratory longitudinal analysis of fecal microbiota shifts in healthy adult dogs during a 90-day dietary trial period. By comparing a control diet with two hempseed-based formulations (fiber-rich by-product vs. fat-rich oil coating), we aimed to characterize formulation-specific trajectories of microbial diversity and taxonomic shifts. This exploratory study provides a framework for generating hypotheses regarding the microbiota-modulating potential of hempseed-based ingredients in canine nutrition, though future studies with compositionally matched diets are needed to confirm specific effects.

## 2. Materials and Methods

### 2.1. Animals and Housing

A cohort of 24 healthy mixed-breed dogs aged 3–6 years and weighing 15–20 kg was randomly selected for this study. The dogs were housed separately in kennels and divided equally by sex (four males and four females) into each group, and all had an appropriate body condition score. A priori power analysis (GPower 3.1.9.4) for the primary outcome (Chao1 alpha diversity) using a repeated-measures within-between interaction [7,17] assumed a medium-to-large effect size (f = 0.4) and a correlation of r = 0.5 among repeated measures [7]. With α = 0.05 and power = 0.95, the minimum sample size was 24 dogs (8 per group). This sample size was calculated specifically for the primary outcome (alpha diversity). Differential abundance analysis of hundreds of taxa (DESeq2) was performed as exploratory to generate hypotheses, not as a confirmatory test, and multiple testing correction (Benjamini–Hochberg FDR) was applied accordingly. During the pre-acclimatization phase, all dogs were fed a control diet for 25 days. An acclimatization period (30 days) followed, during which they were individually housed and fed a control diet tailored to meet their daily metabolizable energy (ME) requirements before the experiment. Health status was monitored throughout this period by a veterinarian through clinical observations, physical examinations, hematological analyses, and blood chemistry profiling to confirm optimal health prior to the start of the experiment. No dog required removal from the study. All procedures complied with institutional guidelines. The study was approved by the Animal Care and Use Committee for Scientific Research ACKU66-VET-080).

### 2.2. Dietary Intervention

Following the acclimatization period, the dogs were randomly assigned to one of three experimental groups (*n* = 8 per group) for a 90-day dietary intervention. All groups received the same nutritionally balanced basal diet formulated to meet the maintenance energy requirements of adult dogs. The three experimental groups were defined as follows: (1) control group (FC): unmodified basal diet.; (2) hemp seed by-product group (FB): diet mixed with hemp seed by-product (hempseed cake). The by-product was prepared as a premix in batches and integrated into the feed formulation at 11%; and (3) hemp seed oil coating group (FO): diet coated with hemp seed oil. The FO was produced by applying 2% hemp seed oil via spray *application* during the cooling phase of feed production.

All formulated diets underwent proximate analysis, which confirmed approximately similar levels of the main nutritional parameters across the three groups. Detailed component percentages and nutritional composition data are provided in Supplementary Table S1, respectively. The daily feed quantities were calculated to fulfill the individual ME requirements of each dog, with each dog receiving a single meal consisting of 300 g per dog per day. Fresh water was provided *ad libitum* throughout the study. Daily feed quantities were calculated in accordance with the AAFCO’s daily energy requirement recommendations, utilizing a factor of 1.6. For dogs weighing 15–20 kg, this resulted in a range of 260–330 g/day. To standardize feeding across groups, all dogs received a fixed ration of 300 g/day, which fell within this calculated range for every dog. Body weight remained stable throughout the study.

### 2.3. Sample Collection

Fecal and blood samples were collected from all dogs at baseline (day 0) and on days 30 and 90 of the dietary intervention period. Fecal score (7-score) was evaluated. Following natural defecation, fecal samples were collected individually from the floor using sterile gloves and a sterile spatula. Samples were placed in sterile labelled bags, transported on ice, and stored at −80 °C. No pooling was performed. No dog experienced diarrhea or received any medication before and during the study. Blood samples (approximately 2 mL) were collected via venipuncture to examine the blood parameters.

### 2.4. DNA Extraction and Quality

DNA was extracted from canine fecal samples using a QIAamp PowerFecal Pro DNA Kit (Qiagen, Hilden, Germany) according to the manufacturer’s protocol, with minor modifications to enhance cell lysis. Briefly, 2 g of feces was suspended in 8 mL of phosphate-buffered saline (PBS, pH = 8) and centrifuged at 12,000× *g* for 5 min. The supernatant was discarded, and the resulting pellet was resuspended in 800 μL of lysis buffer. Subsequently, 1 mL of this suspension was transferred to a PowerBead Pro tube and homogenized using a FastPrep-24 Tissue Homogenizer (MP Biomedicals, Santa Ana, CA, USA) at 6.5 m/s for three cycles, with 5 min incubations on ice between each cycle. Following homogenization, the tubes were centrifuged at 15,000× *g* for 1 min, and the supernatant was collected for subsequent DNA purification according to the manufacturer’s protocol. Extracted DNA was stored at −20 °C until downstream microbiome analysis. DNA quality was assessed by measuring the A260/A280 ratio using a Nanodrop 2000c spectrophotometer (Thermo Fisher Scientific, Waltham, MA, USA); DNA extracts with an A260/A280 ratio between 1.8 and 2.0 were qualified for subsequent analysis.

### 2.5. 16S rRNA Next Gene Sequencing

Polymerase chain reaction (PCR) amplification of the 16S rRNA (V3–V4) region was performed using the universal primers 314F and 805R in a reaction mixture containing a master mix, primers, and diluted DNA samples (25 mg feces). The reactions were performed using a thermocycler (Bio-Rad, Hercules, CA, USA), and agarose gel electrophoresis (Bio-Rad, Hercules, CA, USA) was used to assess the quality of the PCR results. Aliquots (5 μL) of each reaction were analyzed on a 2% (*w*/*v*) agarose gel in 1× TAE buffer using a 100 bp DNA ladder as a reference. The power supply was maintained at a constant voltage of 80 V for 30 min. For library quantification, libraries were constructed, and 16S rRNA gene V3–V4 sequencing was performed using an Illumina NovaSeq 6000 (Illumina Inc., San Diego, CA, USA) after the libraries passed quality inspection.

### 2.6. Bioinformatics

The 16S rRNA gene sequences were processed using bioinformatics pipelines. Paired-end reads were subjected to quality and primer trimming using fastp [18], retaining sequences with a quality score > 15 at the 3′ terminus. Subsequently, high-quality paired-end reads were merged into single reads using FLASH [19], and chimeric sequences were identified and removed using the UCHIME algorithm [20]. The resulting high-quality single reads were clustered into operational taxonomic units (OTUs) at 97% sequence similarity using VSEARCH [21]. Taxonomic assignment was performed using the QIIME2 naïve Bayes classifier (v 2024.10) [22] against the SILVA 99% OTU database v.138 [22], with a confidence threshold of 70%. Alpha diversity metrics, including the Chao1, Shannon, and Simpson indices, were computed using the vegan package (v.2.5.6) [23]. Beta diversity was assessed using a principal coordinate analysis (PCoA) plot based on Bray–Curtis dissimilarity, implemented in the vegan R package. Bacterial community data at the phylum, family, and genus levels were integrated for each dietary group at each time point. Microbial abundance and data visualization were generated using several R packages.

### 2.7. Prevalence Analysis

The prevalence (%) was calculated as the proportion of samples in which each genus was detected. A genus was considered present when it was detected above the predefined threshold (e.g., relative abundance > 0%). Prevalence was computed using the following formula: Prevalence = (Number of samples in which the genus was present/Total number of samples) × 100. Differences in prevalence between groups were assessed using the chi-square test. Fisher’s exact test was applied when the expected cell counts were <5. *p*-values were adjusted for multiple comparisons using the Benjamini–Hochberg false discovery rate (FDR) correction.

### 2.8. Statistical Analysis

All statistical analyses and data visualizations were performed using R (version 4.4.1) [24] and ggplot2 package v3.5.2 [25]. Differential abundance analysis between groups was performed using DESeq2 v1.50.0 [26,27]. Log transformation of microbiome data was applied before using the model, and taxa with very low prevalence were excluded to minimize excess zeros and enhance model robustness. Differential analysis of the microbiome data in terms of % relative abundance between diet groups was conducted using linear mixed-effects models. The models incorporated diet group and treatment day as fixed effects, along with their interaction, whereas individual animals were included as a random effect to account for repeated measurements within subjects. The relative abundance of bacterial taxa was treated as a response variable. Results are presented as least-squares means ± standard error of the mean (SEM) using the ‘emmeans’ package in R Studio. Statistical significance was set at *p* < 0.05. Heatmaps and dendrograms were generated using the ‘pheatmap’ package in R Studio. Factor scores were analyzed using linear mixed-effects models, with day and diet as fixed effects and animal as a random effect. Interaction terms were included to assess the diet-specific temporal responses.

## 3. Results

### 3.1. Dog Health (Fecal Scores and Blood Parameters)

The evaluation of body condition score (BCS), feed intake, fecal score, and blood profiles is presented in Supplementary Tables S2 and S3. No statistically significant differences were identified between the groups in terms of body weight, food consumption, body condition score, or fecal score throughout the experimental period. Additionally, all animals maintained optimal health, with their blood profiles remaining within normal parameters, and none of the dogs exhibited adverse clinical symptoms or notable disturbances in gastrointestinal functions.

### 3.2. Temporal and Dietary Drivers of Alpha Diversity and Community-Level Shifts in Beta Diversity

From the proximate nutritional composition analysis of the diets, all diets had a similar protein content of 26–27%. The hemp seed by-product (FB) group had the highest dietary fiber level (9.06%), whereas the dietary treatment with hempseed oil (FO) group had the highest fat content (16.74%); however, these differences in nutrient composition did not significantly affect the fecal consistency scores or feed intake among the groups.

In this study, sequencing and bioinformatics analysis of the dog microbiota yielded a total of 13,359,534 paired-end sequences, with a minimum of 108,361, a maximum of 308,406, and a median of 203,701 pairs per sample.

**Figure 1:**
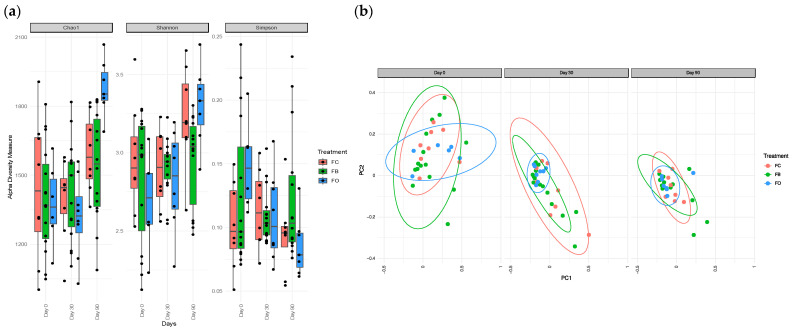
Bacterial diversity of the population. (**a**) Alpha diversity indices (i.e., Chao1, Shannon, and Simpson). (**b**) Beta diversity: Principal Coordinate Analysis (PCoA) plot using Bray–Curtis dissimilarity matrices of the bacterial population in fecal microbiome.

Dietary treatment with hempseed oil (FO) and its by-products (FB) modulated the canine gut microbiota over 90 days. A significant effect of day was observed on species richness (Chao1, *p* < 0.01) and Shannon index (*p* = 0.002) (Table 1; Figure 1a). A significant diet-by-day interaction was also observed for Chao1 (*p* = 0.016). The FO group exhibited the most pronounced increase in both Chao1 (1374.38 on day 0 to 1879.36 on day 90) and Shannon index (2.69 on day 0 to 3.31 on day 90), whereas the changes in the FC and FB groups were more modest. The Simpson index did not differ significantly among the diets or time points, remaining between 0.09 and 0.15 for all groups. This pattern was evident across all dietary groups, suggesting that the observed shifts in microbial diversity were primarily associated with time rather than specific dietary treatments.

Beta diversity analysis based on Bray–Curtis dissimilarity revealed overlapping clusters of microbial communities among the three dietary groups at each time point in the PCoA plot (Figure 1b). However, PERMANOVA indicated a significant effect of the day of the experiment on the overall fecal microbiome structure (*p* = 0.001; Supplementary Table S4), with the community cluster size decreasing from day 0 to day 90. The pairwise beta diversity analysis showed that the community structure on day 0 vs. day 30, day 0 vs. day 90, and day 30 vs. day 90 was significantly different (*p* < 0.01). The patterns among dietary groups indicated a shared temporal progression of gut microbiota during the experimental period.

### 3.3. Compositional Dynamics of the Fecal Microbiome

At the phylum level, Firmicutes was the most abundant, comprising over 72.3% of the sequences, followed by Actinobacteriota (13–17%) (Table 2). A diet-by-day interaction was observed for Actinobacteriota, with the FB group showing the highest relative abundance (17.5%). The relative abundance of Bacteroidota was significantly influenced by day (*p* < 0.05), with the highest levels in the FO group (0.6).

**Figure 2:**
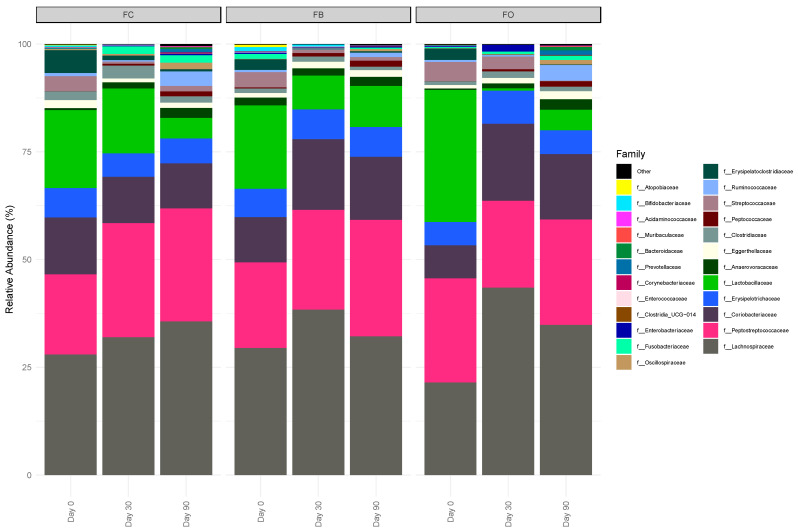
The relative abundance (%) of the bacterial communities at the family level.

At the family level, *Lachnospiraceae* was the most abundant family across all groups. A significant diet-by-day interaction was observed for *Lachnospiraceae* (*p* = 0.01) and *Coriobacteriaceae* (*p* = 0.05) (Table 3; Figure 2). Diet had a significant main effect on the abundance of *Clostridiaceae* (*p* = 0.04) and *Oscillospiraceae* (*p* < 0.01) in the canine gastrointestinal tract. The FC group had the highest relative abundances of *Clostridiaceae* (2.0%) and *Oscillospiraceae* (0.7%). The day of the experiment significantly influenced the abundance of multiple families, including *Lachnospiraceae*, *Coriobacteriaceae*, *Lactobacillaceae*, *Streptococcaceae*, and *Ruminococcaceae* (*p* < 0.05 for all).

**Figure 3:**
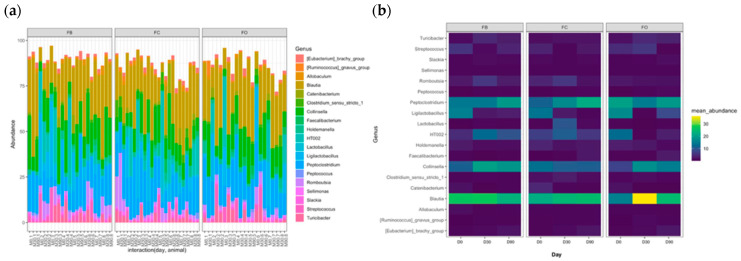

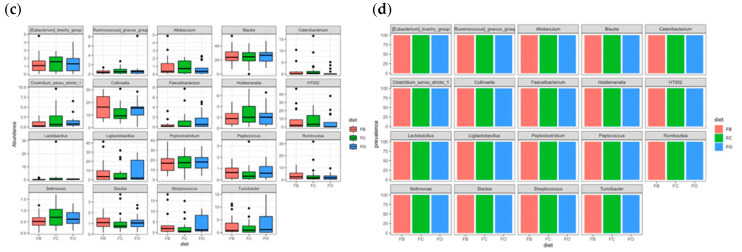
Relative abundance and prevalence of the bacterial communities at the genus level. (**a**) Stacked bar plot of the bacterial community composition per sample. (**b**) Heatmap of the bacterial communities of mean abundance of genera across Day × Diet. (**c**) Boxplot of genus abundance by Day or Diet. (**d**) Prevalence plot of each genus (% of samples).

At the genus level, a high degree of interindividual variation was observed (Figure 3a). *Blautia* was the most abundant genus across all groups, with a mean relative abundance of 24.7% (Figure 3b). Several genera were significantly affected by the day (*p* < 0.05), including *Blautia*, *Peptoclostridium*, *Collinsella*, *Ligilactobacillus*, and *Faecalibacterium* (Supplementary Table S5). A significant diet × day interaction was observed for *Blautia*, *Collinsella*, and *Faecalibacterium*. *Collinsella* abundance was highest in the FB group, whereas *Ligilactobacillus*, *Streptococcus*, and *Turicibacter* abundance were higher in the FO group (Figure 3c). Prevalence analysis revealed a core set of 19 genera that were consistently detected in all samples (Figure 3d). Across all time points, pairwise comparisons between the FB, FC, and FO diets revealed no statistically significant differences (Figure S1).

**Figure 4:**
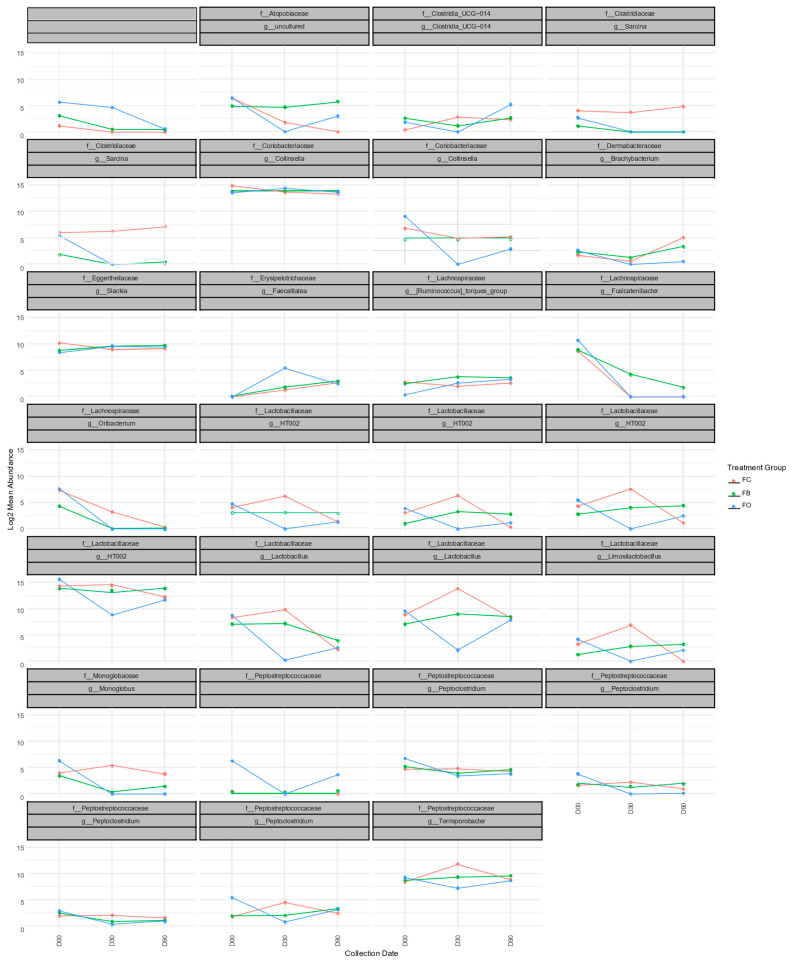
Differences in taxonomic abundance between diet groups on DESeq2 for linear regression, setting *p* = 0.05.

Differential abundance analysis using DESeq2 confirmed significant fluctuations in several taxa over time and between the diets (Figure 4). In the FC and FO groups, the abundance of genera such as *Lactobacillus*, *Ligilactobacillus*, *Monoglobus*, and *Peptoclostridium*, significantly changed from day 0 to day 90. In contrast, the FB group exhibited a more stable abundance of taxa, including *Collinsella*, *HT002*, *Slackia*, and *Peptoclostridium* over the 90-day period.

**Figure 5:**
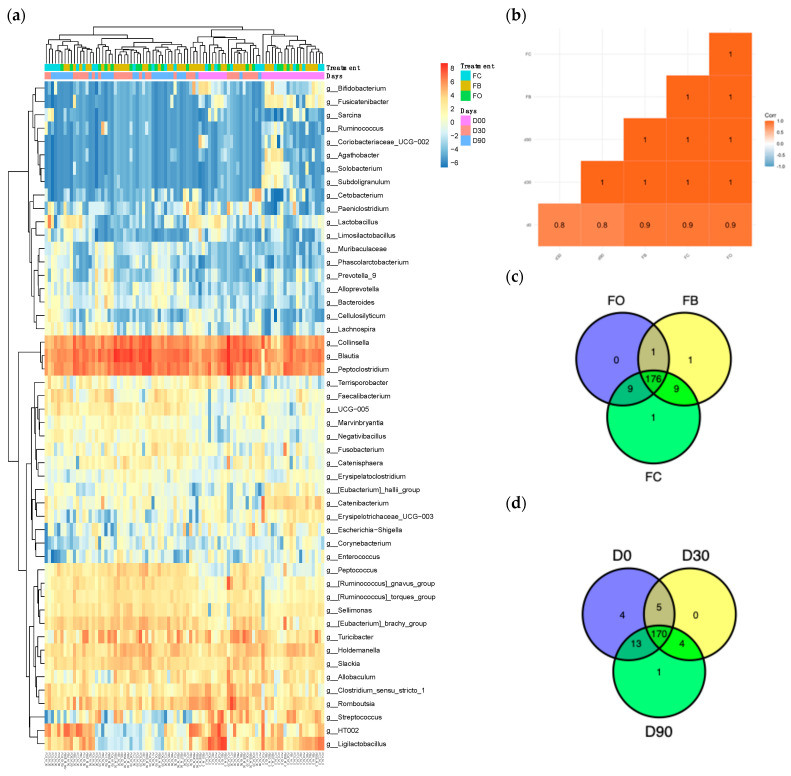
Diet–relation relationship. (**a**) Heatmap and cluster analysis of bacterial microbiome at the genus level in feces. (**b**) Correlation matrix with correlation coefficient between day (day 0, day 30, day 90) and diet treatment (FC, FB, FO) at the genus level (relative abundance > 0.9%) *(p*-value ≤ 0.05). Positive correlations are shown in red. Color intensity is proportional to the correlation coefficients within a correlation group. (**c**) Venn diagram by diet group. (**d**) Venn diagram by day.

The results of the cluster analysis of the bacterial microbiome demonstrated a clear temporal effect (Figure 5a). Abundance profiles on days 30 and 90 were largely grouped into distinct clusters. *Collinsella*, *Blautia*, and *Peptoclostridium* were abundant throughout the study and clustered together. Pearson correlation analysis revealed a strong positive correlation (r = 0.9) between the bacterial community structure in all dietary treatments, indicating a similar bacterial population throughout the study period (Figure 5b). A total of 197 genera were identified in all samples. Among these, 176 genera were consistently observed across different diets, and 170 genera were found across various days within the three groups, indicating the existence of microbiota (Figure 5c,d).

### 3.4. Multivariate Analysis of Temporal and Dietary Shifts

**Figure 6:**
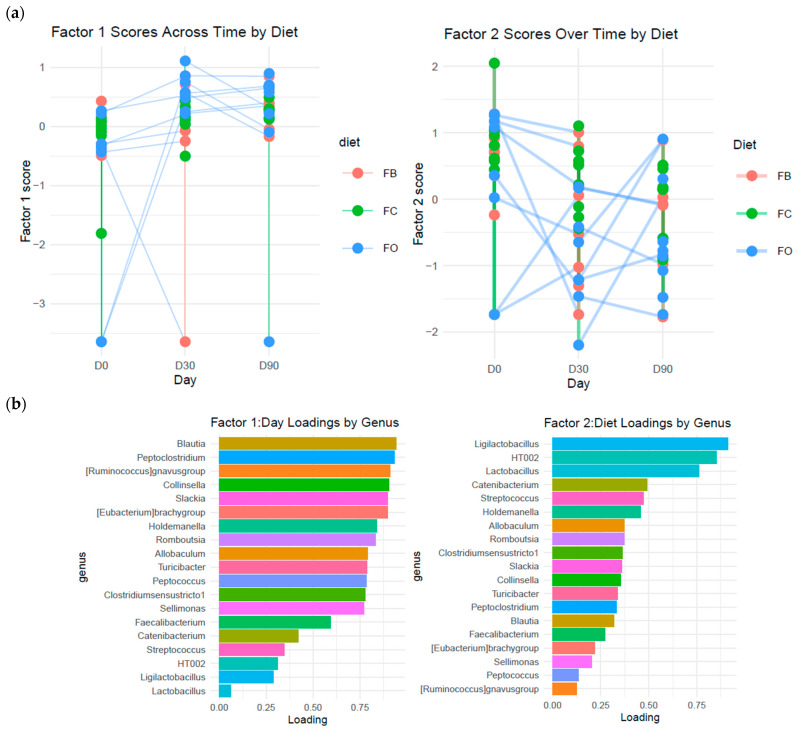
Factor analysis of diet-specific temporal responses. (**a**) Longitudinal changes (factor scores) by factor 1 (day) and factor 2 (diet); (**b**) bar plot of factor loadings at genus level. Factor scores were analyzed using linear mixed-effects models with day and diet as fixed effects and animal as a random effect. |loading| ≥ 0.3 means contribution with positive (+) vs. negative (−) direction of association.

A longitudinal analysis of the factor scores confirmed distinct temporal and diet-related patterns (Figure 6a). Factor 1, which captures the variation associated with time, showed an upward trend from day 0 to day 90, with the FO group displaying the most pronounced increase and greatest inter-individual variability. Factor 2, associated with diet, revealed marked differences between groups, particularly on day 30, when the FO group showed a distinct shift toward negative scores, indicating a strong diet-specific response.

The analysis of the factor loadings identified the key genera driving these patterns (Figure 6b). Factor 1 (day) was primarily driven by high positive loadings (>0.75) from *Blautia*, *Peptoclostridium*, *Ruminococcus gnavus* group, *Collinsella*, *Slackia*, and Slackia, indicating a shared temporal dynamic among these taxa. Factor 2 (diet) showed strong positive loadings (>0.75) for *Ligilactobacillus*, *GHT002*, and *Lactobacillus*, identifying them as key taxa differentiating the dietary groups.

## 4. Discussion

This longitudinal investigation of canine fecal microbiota over a 90-day dietary intervention with hempseed formulations revealed a complex ecological maturation process. Our findings demonstrate that while the fecal microbiome of healthy dogs exhibits significant temporal shifts under standardized conditions, the introduction of hempseed by-product (FB) and hempseed oil (FO) exerts distinct selective pressures that enhance microbial diversity without disrupting the ecological stability of the host animal. The principal findings revealed that both hempseed-based diets modulated the canine gut microbiota, with the most pronounced effects observed in the FO group. Specifically, FO supplementation led to the greatest increase in alpha diversity and induced distinct shifts in several bacterial taxa, whereas the FB group exhibited a more moderate response and greater stability in certain communities. These results suggest that hempseed, particularly its oil fraction, exerts health effects in dogs, although the specific formulation dictates the nature and extent of microbial modulation [28].

### 4.1. Hempseed Oil-Formulated Enhances Gut Microbial Diversity Through Ecological Maturation

A significant increase in alpha diversity metrics (Chao1 and Shannon indices) from day 0 to day 90 was observed across all groups (Table 1), indicating that the standardized housing and nutritionally balanced diets themselves promote ecological maturation of the gut microbiota over time. This background temporal drift is consistent with previous reports of microbial succession under controlled conditions [2,29]. However, the magnitude and trajectory of this diversification contributed to the diet-related alteration of the communities [30,31]. For species richness (Chao1), a significant diet-by-day interaction (*p* = 0.016) demonstrated that the FO group’s increase was statistically distinguishable from and substantially larger than the increase observed in the FC group. For the Shannon index, the diet-by-day interaction did not reach significance (*p* = 0.092), and the increase in FO was comparable to that in FC after accounting for baseline differences. Thus, while background ecological maturation occurred across all groups, hempseed oil supplementation specifically enhanced species richness beyond this baseline drift. Enhanced microbial diversity, particularly richness, is often associated with gut ecosystem stability [2,29].

The pronounced effect of the FO diet, which had the highest fat content (16.74%), on diversity may be attributed to its high fatty acid content [6,8]. Hempseed oil is known to contain high concentrations of polyunsaturated fatty acids (PUFAs), which have been reported to provide novel metabolic niches that can support subdominant taxa [32,33]; However, the specific fatty acid composition of the experimental diets was not quantified in this study; therefore, the precise mechanisms underlying the observed increase in species richness remain to be determined. In contrast, the fiber-rich hemp by-product diet (FB) had a more muted effect on diversity, possibly due to the complex interplay between its insoluble fiber components and the existing microbial community, which could favor specific fibrolytic bacteria without drastically increasing overall richness [34]. The significant diet-by-day interaction for Chao1 (*p* = 0.016) underscores that diet influenced temporal diversity trajectories, though similar shifts in the control group indicate that other factors (e.g., environment, housing) also contributed [31,35].

Beta diversity analysis further corroborated this temporal progression. The highly significant effect of day on community structure (PERMANOVA, *p* = 0.001) and the contraction of microbial clusters by day 90, as visualized in the PCoA plot (Figure 1b), reflected a reduction in inter-individual variance in the control group. This stabilization is a critical finding, indicating that the gut ecosystem reaches a new equilibrium over a 90-day period in response to novel functional ingredients [36], as indicated by the significant difference at each time point (Table S4). The convergence of microbial communities toward a more homogeneous structure suggests that while dietary interventions likely contributed, the convergence across all groups, including control, suggests that common environmental or management factors also played a role [36].

### 4.2. Divergent Microbial Communities Across Hempseed Fractions

Differential abundance analysis and factor loading elucidated clear taxonomic signatures associated with each hempseed fraction. At the phylum level, the significant diet-by-day interaction for Actinobacteriota (*p* = 0.05), with the highest relative abundance in the FB group (17.5%), was particularly noteworthy. This phylum contains beneficial genera such as *Bifidobacterium* and *Collinsella*. The observed enrichment of *Collinsella* specifically within the FB group (Figure 3c) aligns with these findings and implies that the higher relative abundance of *Collinsella* in the FB group may relate to the by-product of fiber content, as some *Collinsella* species ferment carbohydrates [37,38]. *Collinsella* species are recognized as carbohydrate fermenters that contribute to the conversion of dietary fibers into short-chain fatty acids (SCFAs). An increase in their abundance has been shown in response to dietary fiber intake, potentially leading to improved metabolic outcomes [37,38]. Conversely, some human studies indicate that low dietary fiber consumption may also elevate *Collinsella* levels, thereby altering the overall fermentation patterns of the gut microbiota [39,40]. However, our results contrast with those of a previous study on dogs, which reported that *Collinsella intestinalis* was among the predominant species in the fecal microbiomes of animals fed a low-protein, low-fiber diet supplemented with a yeast probiotic [41]. Notably, *Collinsella* is also recognized as a member of the microbiome in healthy dogs and is enriched in dogs fed a kibble-only diet [12]. This contextualizes the observed stability of *Collinsella* in the FB group over the 90 days; as indicated by DESeq2 analysis, this persistence may indicate that the hempseed by-product provides a consistent fermentable substrate or that specific fiber components in the hempseed by-product may preferentially support this microbial population.

At the family level, a significant diet effect was observed on *Clostridiaceae* and *Oscillospiraceae*, with higher abundance in the feces of the FC and FO groups than in those of the FB group. This finding is consistent with that of an earlier study that reported that *Clostridiaceae* are predominant in dogs fed a commercial diet [13]. Interestingly, an earlier study in mice showed a higher abundance of *Clostridiaceae* in response to a hempseed-based diet [18]. While some members of these families are associated with negative health outcomes, others are important butyrate producers of the gut. Nevertheless, the observed shift in these families is noteworthy, as *Clostridiaceae* abundance is positively associated with dietary protein and fat and negatively associated with fiber in dogs [42]. Consistent with this, the fiber-rich FB group showed lower *Clostridiaceae* abundance than the FC and FO groups.

The observed suppression of these families in the FB group suggests that hempseed by-products may promote competitive exclusion, thereby favoring the growth of beneficial fermenters such as *Coriobacteriaceae*. This aligns with previous research indicating that fiber-rich diets can increase the abundance of *Coriobacteriaceae* [43]. There was a significant increase in butyrate-producing families *Lachnospiraceae* and *Ruminococcaceae* over time (day effect, *p* < 0.01 for both), though direct SCFA measurement is required to confirm enhanced functional capacity. Their increase over time in all groups suggests that the functional capacity of the microbiome, particularly for producing short-chain fatty acids (SCFAs), can shift independently of other taxonomic changes. As key SCFA producers, these probiotic families play a crucial role in promoting canine intestinal health [2].

Genus-level analysis provides the most detailed insights. The high prevalence and abundance of *Blautia* in all groups align with its status as a core canine gut microbe [2,3]. A significant diet-by-day interaction (*p* < 0.05) was characterized by a peak at day 30 in the FB and FO groups, followed by a decline at day 90. This temporal pattern of increasing diversity and community convergence is consistent with ecological maturation, although comparable patterns in the control group point to contributions from environmental and management factors. This pattern is supported by previous research, including a human study in which daily omega-3 consumption led to a significant increase in *Blautia* abundance over a short period [44]. This transient stimulatory effect suggests an initial adaptive fermentation response to novel substrates, which eventually stabilizes as the community optimizes its functional capacity, a pattern consistent with the potential probiotic properties of *Blautia* [3,45]. Furthermore, dietary patterns beyond supplementation have been shown to influence the microbiota; for instance, alternating diets have been associated with enhanced abundance of *Blautia* and *Ruminococcus* compared to a conventional diet [45].

The hempseed oil intervention (FO) was characterized by the specific enrichment of *Ligilactobacillus* and *HT002* genus. Factor 2 (diet) showed strong positive loadings (>0.75) for *Ligilactobacillus*, *GHT002*, and *Lactobacillus*, identifying these as key taxa differentiating the dietary groups. Validation in larger independent cohorts is needed to confirm their potential as diet-responsive biomarkers. *Ligilactobacillus* is a well-documented producer of lactic acid with established probiotic properties, including support for gut barrier integrity. The enrichment of this genus in the FO group suggests that the unique fatty acid profile of the oil may confer a selective advantage, a finding of particular interest given that *Ligilactobacillus* spp. have been recognized as promising candidates for novel probiotic formulations for dogs [46].

The concurrent identification of the *HT002* genus adds taxonomic nuance. Within the SILVA v138 database, the *HT002* designation often indicates sequences with low specificity for *Limosilactobacillus* when using the V3–V4 hypervariable region. This suggests that the observed enrichment may indicate a broader and selective promotion of *Lactobacillaceae* family members by hempseed oil. This effect could be mediated through mechanisms such as the modulation of lipid-mediated signaling pathways or diversification of the bile acid pool.

### 4.3. Ecological Maturation of the Canine Gut Microbiota

The persistence of several genera across all samples indicates relative compositional stability within this cohort. However, without a validated dysbiosis index or functional assessment, we cannot conclude eubiosis versus dysbiosis. Furthermore, this cohort-specific finding does not define a universal canine microbiota. The identification of 19 dominant genera common to the three distinct dietary groups supports the notion of a stable and foundational canine gut microbiota that remains largely consistent despite dietary variations [2,3,7].

Factor analysis elegantly summarized the primary drivers of microbial variation. Factor 1 (day) captured a temporal signature driven by high positive loadings (>0.75) from genera such as *Blautia*, *Peptoclostridium*, *Ruminococcus gnavus* group, *Collinsella*, and *Slackia*, indicating that these taxa are highly responsive to the passage of time and dietary adaptation. Factor 2 (diet) showed strong positive loadings (>0.75) for *Ligilactobacillus*, *GHT002*, and *Lactobacillus*, identifying these as key taxa differentiating the dietary groups. The differential abundance analysis using DESeq2 further highlighted the dynamic nature of these changes, with the FB group exhibiting a more stable community composition over 90 days compared to the FC and FO groups. This stability could be interpreted as the FB diet being more closely aligned with the baseline microbiota’s metabolic capabilities, or it could indicate that in this case, the fiber component promotes a community that is less susceptible to temporal fluctuation.

### 4.4. Clinical and Methodological Implications

The application of linear mixed-effects modeling and factor analysis in this study provided a framework for partitioning the variance attributable to time and dietary factors. By treating individual dogs as random effects, we accounted for inter-individual variability. The 90-day longitudinal design proved essential, as many significant community shifts were observed, and the community structure changed at each time point within 90 days. This underscores the necessity of extended temporal sampling in dietary intervention studies to distinguish true ecological stabilization from transient, adaptive responses. Furthermore, this study may support previous research showing that hempseed oil supplementation at 1–2% positively affects nutrient digestibility, blood metabolism, immunity, and antioxidant capacity in dogs [47]. However, these factors should be included in future experiments.

### 4.5. Study Limitations and Future Directions

This study had certain limitations. First, while the sample size (n = 8 per group) was adequately powered for the primary outcome (alpha diversity), it was not powered for confirmatory differential abundance analysis of hundreds of taxa. Therefore, taxon-level findings should be considered exploratory and hypothesis-generating, requiring validation in larger independent cohorts. Second, the hempseed-based formulations differed from the control not only in hempseed inclusion but also in macronutrient composition. Consequently, the observed microbiome shifts cannot be attributed specifically to hempseed derivatives independent of changes in dietary fiber and fat. Third, 16S rRNA gene sequencing provides compositional data but not direct functional insights. Direct measurement of SCFAs, inflammatory markers, or metagenomics would be needed to confirm functional outcomes [48].

## 5. Conclusions

This 90-day study demonstrated that dietary supplementation with hempseed by-products and oil modulates the canine fecal microbiome in distinct formulation-specific ways. While background ecological maturation occurred across all groups, the oil formulation specifically enhanced species richness beyond this baseline drift, and both supplements induced distinct taxon-specific shifts: the fiber-rich by-product selectively enriched *Actinobacteriota* (notably *Collinsella*) and the oil favoring *Lactobacillaceae* members (including *Ligilactobacillus*). The persistence of some bacterial groups confirmed that these shifts in compositional changes did not result in a major loss of community stability, thereby supporting the potential for health effects as functional ingredients in canine nutrition. Future studies should integrate these taxonomic findings with metabolomic profiles and clinical biomarkers to further elucidate the host–microbe interactions driven by industrial hemp.

## Figures and Tables

**Table 1:** Alpha diversity of the bacterial community in the fecal sample of healthy canines administered a diet incorporating hemp from day 0 to day 90.

Parameter	Alpha Diversity Index								
	Chao1			Shannon			Simpson		
	Day 0	Day 30	Day 90	Day 0	Day 30	Day 90	Day 0	Day 30	Day 90
FC	1438.32	1393.66	1596.74	2.96	2.9	3.23	0.1	0.11	0.09
FB	1347.19	1553.27	1469.55	2.81	2.9	2.94	0.13	0.11	0.13
FO	1374.38	1318.37	1879.36	2.69	2.82	3.31	0.15	0.11	0.09
*p*-Value									
Diet	0.536			0.293			0.287		
Day	<0.01			0.002			0.23		
Diet x Day	0.016			0.092			0.107		

**Table 2:** The most abundant phyla (>0.01% relative abundance of all reads) in healthy dogs fed a hemp diet.

Taxonomy	Dietary Treatment			SEM	*p*-Value		
	FC	FB	FO		Diet	Day	Diet x Day
Firmicutes	84.7	77.8	72.3	4.25	0.12	0.92	0.34
Actinobacteriota	13.5	17.5	13.4	1.77	0.16	0.07	0.05
Fusobacteriota	1.1	0.1	0.5	0.34	0.12	0.30	0.42
Proteobacteria	0.2	0.4	0.6	0.37	0.49	0.71	0.48
Bacteroidota	0.5	0.1	0.6	0.27	0.29	0.02	0.35
Chloroflexi	0.01	0.01	0.00	0.01	0.48	0.19	0.55

**Table 3:** The most abundant family (>0.1% relative abundance of all reads) in healthy dogs fed the hemp diet.

Taxonomy	Dietary Treatment			SEM	*p*-Value		
	FC	FB	FO		Diet	Day	Diet x Day
*Lachnospiraceae*	31	30.7	29.9	2.82	0.95	0.03	0.01
*Peptostreptococcaceae*	23.4	20.8	20.3	2.19	0.56	0.11	0.70
*Coriobacteriaceae*	11.8	15.8	12.2	1.61	0.12	0.02	0.05
*Lactobacillaceae*	13.8	13.1	9.2	3.44	0.55	0.02	0.50
*Erysipelotrichaceae*	5.9	5.7	5.5	0.86	0.95	0.81	0.59
*Streptococcaceae*	1.8	2.6	2.1	0.80	0.75	0.01	0.51
*Anaerovoracaceae*	1.4	1.3	1.1	0.17	0.73	<0.01	0.91
*Eggerthellaceae*	1.4	1.4	1.2	0.20	0.57	0.33	0.03
*Clostridiaceae*	2.0	0.7	1.1	0.37	0.04	0.25	0.93
*Erysipelatoclostridiaceae*	2.4	1.2	0.7	0.62	0.14	<0.01	0.50
*Ruminococcaceae*	1.5	0.6	1.3	0.33	0.14	<0.01	0.19
*Fusobacteriaceae*	1.1	0.1	0.5	0.34	0.08	0.22	0.59
*Peptococcaceae*	0.5	0.5	0.5	0.09	0.98	<0.01	0.81
*Oscillospiraceae*	0.7	0.2	0.4	0.11	<0.01	<0.01	0.11
*Enterobacteriaceae*	0.2	0.4	0.6	0.36	0.71	0.47	0.48
*Bifidobacteriaceae*	0.09	0.06	0.01	0.05	0.52	0.05	0.69
*Prevotellaceae*	0.28	0.05	0.33	0.14	0.34	0.01	0.39
*Atopobiaceae*	0.04	0.04	0.01	0.02	0.64	0.49	0.35

## Data Availability

The data presented in this study are openly available in [NCBI BioProject database (PRJNA1445391)] [https://www.ncbi.nlm.nih.gov/bioproject/] [SRR37899524-37899612] (accessed on 1 April 2026).

## Supplementary Materials

The following supporting information can be downloaded at https://www.mdpi.com/article/10.3390/vetsci13060534/s1, Table S1: Component percentages and nutritional composition of hemp diet; Table S2: An assessment of body condition score (BCS), feed intake, and fecal score with results summarized using means and standard deviations (SD); Table S3: An assessment of blood hematology and chemistry with results summarized using means and standard deviations (SD); Table S4: Analysis of PERMANOVA (Bray–Curtis); Table S5: The most abundant genera (>0.1% relative abundance of all reads) in healthy dogs fed the hemp diet; Figure S1: pairwise contrast plots for the three diets (FB, FC, FO) across three timepoints (D0, D30, D90). (a) FDR method (adjust = “fdr”); (b) Tukey method (adjust = “tukey”).
