# Supplementary material for: Longitudinal Assessment of the Canine Fecal Microbiota in Response to Dietary Hempseed By-Product and Oil: A 90-Day Nutritional Intervention Study
Source: Vet Sci. 2026 May 29;13(6):534. doi: 10.3390/vetsci13060534 (PMC13308462; doi:10.3390/vetsci13060534)
Supplement: Supplementary file 1 [file vetsci-13-00534-s001.zip › vetsci-4317722-supplementary.pdf]

Article

Longitudinal Assessment of the Canine Fecal Microbiota in Response to Dietary Hempseed By-Product and Oil: A 90-Day Nutritional Intervention Study

Jutamat Klinsoda [1], Sasithorn Limsuwan [1,2], Witchayaporn Sornard [3], Pattarawadee Thamsatit [4] and Natthasit Tansakul [2,5,*]

[1] Institute of Food Research and Product Development, Kasetsart University, Bangkok 10900, Thailand; ifrjmk@ku.ac.th (J.K.); sasithorn.limsu@ku.th (S.L.)
[2] Special Research Incubator Unit for Cannabis-Hemp and Phytochemicals in Veterinary Medicine, Faculty of Veterinary Medicine, Kasetsart University, Bangkok 10900, Thailand
[3] Graduate Program in Animal Health and Biomedical Sciences, Faculty of Veterinary Medicine, Kasetsart University, Bangkok 10900, Thailand; witchayaporn.sor@ku.th
[4] Kasetsart Veterinary Teaching Hospital, Faculty of Veterinary Medicine, Kasetsart University, Bangkok 10900, Thailand; gracepatt789@gmail.com
[5] Department of Pharmacology, Faculty of Veterinary Medicine, Kasetsart University, Bangkok 10900, Thailand
* Correspondence: natthasit.t@ku.th

Academic Editor: Jianzhu Liu

Received: 28 April 2026
Revised: 22 May 2026
Accepted: 26 May 2026
Published: 29 May 2026

Copyright: © 2026 by the authors. Submitted for possible open access publication under the terms and conditions of the Creative Commons Attribution (CC BY) license.

Supplementary file

Table S1: Component percentages and nutritional composition of hemp diet.

Ingredient	Components (%)		
	Control (FC)	Hempseed byproduct (FB)	Hempseed oil (FO)
Cassava	31.41	30.41	31.41
Soybean meal	10	10	10
Cassava meal	10	-	10
Poultry meal	10	10	10
Meat & Bone meal	15	15	15
Tuna meal	8	8	8
Cellulose fiber	1	1	1
Hemp byproduct	-	11	-
Premix	2.01	2.01	2.01
Poultry fat	4.5	4.5	4.5
Beef tallow	3.5	3.5	2
Marine fish oil	1.5	1.5	1
Hempseed oil	-	-	2
Liquid palatants	2.5	2.5	2.5
Powder palatants	0.5	0.5	0.5
Preservative	0.08	0.08	0.08
TOTAL	100	100	100
Proximate nutritional composition (%)			
Moisture	7.29	6.91	3.48
Protein	26.1	27.26	26.77
Fat	13.35	12.57	16.74
Fiber	5.95	9.06	6.82
Ash	13.55	12.35	13.84

Methodology: Moisture (AOAC official method -2016-930.15), Protein (AOAC official method -2016-2001.11), Fat (AOAC official method -2016-2003.05), Fiber (AOCS approved procedure Ba 6a-05), Ash (AOCS official method -2016-942.05)

Table S2 An assessment of body condition score (BCS), feed intake, and fecal score with results summarized using means and standard deviations (SD).

		FC		FB		FO		*p*-value
BCS		Mean	SD	Mean	SD	Mean	SD	
(9-score)	Day-0	4.12	0.60	4.50	0.50	4.12	0.78	0.385
	Day-45	4.13	0.60	4.37	0.48	4.25	0.66	
	Day-90	4.25	0.66	4.25	0.43	4.37	0.68	
Feed intake (g/day)								
	Day (0-3)	268.54	30.05	262.29	24.98	263.75	26.30	0.066
	Day (44-46)	269.58	30.00	260.42	11.22	267.50	20.69	
	Day (88-90)	272.92	14.29	262.71	27.82	273.75	10.96	
Fecal score								
(7-score)	Day (0-3)	2.33	0.56	2.29	0.55	2.38	0.58	0.095
	Day (44-46)	2.04	0.46	2.42	0.50	2.25	0.53	
	Day (88-90)	2.25	0.53	2.42	0.50	2.50	0.51	

BCS was assessed using the 9-point Laflamme scale (1 = emaciated, 4-5 = ideal, 9 = obese). Fecal score was assessed using a 7-point scale (1 = very hard/dry pellets, 2 = hard/dry, 3 = firm/dry, 4 = firm/moist, 5 = soft/moist, 6 = loose/pudding-like, 7 = watery diarrhea). Feed intake is reported in grams per day (g/day).

Table S3 An assessment of blood hematology and chemistry with results summarized using means and standard deviations (SD).

Parameter (Abbreviation; normal range)	Date	FC Mean ± SD	FB Mean ± SD	FO Mean ± SD	*P*-value
Hemoglobin HGB (11.9-18.9 g/dL)	Day 0	15.04 ± 1.12	13.71 ± 2.45	13.26 ± 1.47	0.139
	Day 30	13.53 ± 1.44	14.09 ± 1.94	14.03 ± 1.77	0.778
	Day 90	16.20 ± 1.50	15.21 ± 2.01	14.10 ± 0.82	0.071
Hematocrit HCT (35-57 %)	Day 0	44.53 ± 3.27	41.03 ± 6.26	39.68 ± 4.10	0.130
	Day 30	40.05 ± 3.80	43.25 ± 5.47	41.49 ± 4.39	0.396
	Day 90	47.93 ± 4.09	46.04 ± 5.15	41.80 ± 2.49	0.043
Mean corpuscular volume MCV (66-77fL)	Day 0	63.68 ± 2.81	64.25 ± 4.60	65.49 ± 4.51	0.664
	Day 30	65.53 ± 4.60	67.13 ± 5.54	64.03 ± 3.04	0.405
	Day 90	64.01 ± 2.56	66.33 ± 3.94	67.45 ± 4.27	0.208
Mean corpuscular hemoglobin MCH (21-26.2pg)	Day 0	21.51 ± 0.93	21.35 ± 1.17	21.88 ± 1.53	0.690
	Day 30	22.13 ± 1.69	21.80 ± 1.41	21.61 ± 0.99	0.761
	Day 90	21.63 ± 0.85	21.86 ± 1.16	22.77 ± 1.73	0.240
Mean corpuscular hemoglobin concentration MCHC(21-26.2g/dL)	Day 0	33.79 ± 0.52	33.29 ± 1.30	33.41 ± 1.02	0.589
	Day 30	33.74 ± 1.06	32.54 ± 0.79	33.76 ± 1.16	0.040
	Day 90	33.80 ± 0.55	33.00 ± 0.88	33.75 ± 0.91	0.108
Mean Platelet Volume MPV (8.7-13.2fL)	Day 0	10.63 ± 1.14	11.53 ± 1.26	11.71 ± 1.18	0.176
	Day 30	11.16 ± 1.16	10.84 ± 1.44	9.81 ± 1.40	0.134
	Day 90	9.81 ± 1.25	10.75 ± 1.53	10.95 ± 0.90	0.218
Plateletcrit PCT (0.14-0.46%)	Day 0	0.15 ± 0.06	0.16 ± 0.09	0.20 ± 0.10	0.581
	Day 30	0.19 ± 0.09	0.19 ± 0.08	0.20 ± 0.05	0.962
	Day 90	0.21 ± 0.05	0.16 ± 0.08	0.17 ± 0.10	0.435
Reticulocyte percent RET (0-1%)	Day 0	0.46 ± 0.20	1.24 ± 1.36	0.72 ± 0.48	0.197
	Day 30	0.56 ± 0.48	1.11 ± 1.73	0.30 ± 0.14	0.300
	Day 90	0.39 ± 0.20	0.65 ± 0.49	0.69 ± 0.55	0.353
Plasma proteins PP (6-7.5 g/L)	Day 0	7.25 ± 0.87	7.95 ± 1.02	7.15 ± 0.98	0.215
	Day 30	7.65 ± 1.09	8.13 ± 1.53	7.03 ± 0.70	0.187
	Day 90	6.85 ± 0.60	7.90 ± 1.43	7.53 ± 0.99	0.165
Erythrocytes RBC (4.95 - 7.87 10^ 6/uL)	Day 0	6.99 ± 0.37	6.43 ± 1.18	6.08 ± 0.74	0.115
	Day 30	6.14 ± 0.78	6.49 ± 1.09	6.50 ± 0.82	0.673
	Day 90	7.49 ± 0.66	7.00 ± 1.17	6.22 ± 0.61	0.046
White blood cell count WBC (5-14.1 10^ 3/uL)	Day 0	11.38 ± 1.61	10.96 ± 2.93	13.08 ± 2.04	0.163
	Day 30	12.88 ± 1.73	9.98 ± 3.83	11.77 ± 2.12	0.124
	Day 90	11.92 ± 3.31	10.23 ± 3.76	13.22 ± 1.22	0.221
Percent Neutrophil count (NEUT %) NEUT (55-75%)	Day 0	54.56 ± 6.58	56.83 ± 6.32	55.19 ± 8.17	0.806
	Day 30	51.95 ± 6.60	52.53 ± 4.89	53.15 ± 6.51	0.925
	Day 90	56.01 ± 7.95	53.15 ± 8.84	53.30 ± 5.34	0.719
Percent Lymphocyte count LYMPH (13-30%)	Day 0	24.80 ± 8.66	28.13 ± 8.53	23.85 ±8.61	0.588
	Day 30	27.89 ± 8.99	27.78 ± 7.63	23.16 ± 5.72	0.379
	Day 90	23.61 ± 7.62	28.96 ± 7.81	28.63 ± 9.69	0.383

Parameter (Abbreviation; normal range)	Date	FC	FB	FO	*P*-value
		Mean ± SD	Mean ± SD	Mean ± SD	
Percent Monocyte count MONO (2-10%)	Day 0	5.14 ± 1.72	5.36 ± 1.99	4.86 ± 0.93	0.826
	Day 30	5.11 ± 0.45	6.81 ± 3.00	5.64 ± 1.33	0.215
	Day 90	5.23 ± 0.99	6.14 ± 2.73	3.97 ± 1.52	0.141
Percent Eosinophil count EO (0-9%)	Day 0	15.30 ± 5.91	9.43 ± 5.46	15.89 ± 7.59	0.106
	Day 30	14.93 ± 5.75	12.31 ± 5.15	17.91 ± 7.54	0.222
	Day 90	15.05 ± 5.36	11.14 ± 3.80	13.80 ± 5.75	0.299
Percent Basophil count BASO (0-1%)	Day 0	0.20 ± 0.12	0.26 ± 0.18	0.21 ± 0.06	0.614
	Day 30	0.14 ± 0.05	0.66 ± 1.04	0.16 ± 0.08	0.219
	Day 90	0.10 ± 0.08	0.61 ± 0.65	0.13 ± 0.08	0.035
Blood urea nitrogen BUN (8-28 mg%)	Day 0	18.63 ± 2.67	16.75 ± 3.41	21.00 ± 8.52	0.323
	Day 30	13.75 ± 3.49	15.25 ± 3.41	12.50 ± 2.07	0.222
	Day 90	14.75 ± 2.71	17.63 ± 3.96	16.50 ± 2.66	0.225
Creatinine CRE (0.50-1.70 mg%)	Day 0	0.97 ± 0.15	1.07 ± 0.20	1.03 ± 0.17	0.852
	Day 30	0.98 ± 0.10	1.13 ± 0.29	0.96 ± 0.19	0.687
	Day 90	1.00 ± 0.13	1.07 ± 0.21	0.98 ± 0.10	0.906
Cholesterol CHOL (136-392 mg/dL)	Day 0	167.50 ± 13.72	151.38 ± 36.77	152.88 ± 28.00	0.454
	Day 30	145.00 ± 20.23	136.88 ± 26.84	148.38 ± 17.55	0.567
	Day 90	137.88 ± 13.95	140.50 ± 21.75	148.33 ± 17.03	0.554
Triglycerides TRIG (23-102 mg/dL)	Day 0	53.13 ± 11.05	47.50 ± 7.71	49.25 ± 16.02	0.641
	Day 30	49.25 ± 8.92	45.50 ± 10.78	51.13 ± 14.38	0.065
	Day 90	42.75 ± 12.86	40.50 ± 9.21	43.67 ± 5.20	0.826
Total protein (TP) TP (5.4-7.5 g/dL)	Day 0	6.65 ± 0.79	7.20 ± 1.08	6.55 ± 0.80	0.316
	Day 30	6.70 ± 0.81	7.40 ± 1.28	6.45 ± 0.76	0.158
	Day 90	6.19 ± 0.48	7.36 ± 1.18	7.10 ± 0.79	0.037
Albumin ALB (2.3-3.1g/dL)	Day 0	2.90 ± 0.27	2.80 ± 0.59	2.79 ± 0.30	0.840
	Day 30	2.80 ± 0.44	2.81 ± 0.52	2.74 ± 0.28	0.932
	Day 90	2.91 ± 0.22	2.95 ± 0.50	2.85 ± 0.23	0.872
Aspartate aminotransferase AST (13-15 U/L)	Day 0	35.88 ± 7.70	34.63 ± 14.18	31.63 ± 9.32	0.723
	Day 30	35.13 ± 19.42	32.63 ± 12.45	33.13 ± 6.31	0.929
	Day 90	32.13 ± 4.73	30.88 ± 12.79	35.17 ± 15.22	0.781
Alanine aminotransferase ALT (10-109 U/L)	Day 0	41.75 ± 33.71	22.00 ± 9.20	42.00 ± 51.90	0.459
	Day 30	53.88 ± 84.43	18.63 ± 7.23	31.38 ± 12.78	0.371
	Day 90	33.63 ± 15.41	19.13 ± 8.10	61.17 ± 90.97	0.286
Alkaline phosphatase (ALP) ALP (8-76 U/L)	Day 0	23.38 ± 10.64	44.13 ± 57.66	28.50 ± 8.47	0.463
	Day 30	24.75 ± 13.45	34.63 ± 43.66	19.13 ± 9.49	0.518
	Day 90	18.88 ± 10.76	26.13 ± 26.57	21.17 ± 9.00	0.719

Table S4 Analysis of PERMANOVA (Bray-Curtis)

Term	Df	Sum of SQS	R^2	Statistic	*P*-value	Pairwise of DAY_collect at *P* = 0.001		
DAY_collect	2	1.5407	0.1299	6.4148	0.001	Comparison	P.value	P.adjust
GROUP_treatment	2	0.2265	0.01909	0.9429	0.461	0 vs 30	0.001	0.003
Residual	84	10.0875	0.85047			0 vs 90	0.001	0.003
Total	88	11.8612	1			30 vs 90	0.014	0.028

Table S5 The most abundant genera (> 0.1% relative abundance of all reads) in healthy dogs fed hemp diet

Taxonomy	Dietary treatment			SEM	*P*-value		
	FC	FB	FO		Diet	Day	Diet*Day
Blautia	23.8	24.7	23.2	2.43	0.90	0.04	0.01
Peptoclostridium	16.8	15.4	15.7	1.74	0.82	0.04	0.51
Collinsella	11.8	15.8	12.2	1.61	0.12	0.03	0.05
HT002	6.6	6.4	4.0	2.12	0.59	0.67	0.32
Ligilactobacillus	5.7	6.4	5.0	1.66	0.82	<0.01	0.95
Romboutsia	3.7	3.6	1.9	0.95	0.31	0.24	0.86
Turicibacter	2.0	2.6	2.8	0.69	0.66	0.12	0.90
Holdemanella	2.7	1.9	1.8	0.35	0.17	0.04	0.79
Streptococcus	1.8	2.6	2.1	0.81	0.77	0.01	0.51
Clostridium_sensu_stricto_1	1.9	0.7	1.1	0.37	0.07	0.22	0.92
Slackia	1.1	1.1	1.0	0.15	0.66	0.05	0.07
Catenibacterium	1.6	0.9	0.5	0.50	0.31	0.00	0.58
Allobaculum	0.9	0.9	0.6	0.19	0.44	0.48	0.13
Faecalibacterium	1.0	0.3	1.0	0.27	0.10	<0.01	0.01
Peptococcus	0.5	0.5	0.5	0.10	0.98	<0.01	0.82
[Ruminococcus]_gnavus_group	0.7	0.4	0.8	0.20	0.44	0.02	0.70
Sellimonas	0.7	0.5	0.6	0.08	0.17	0.30	0.67
Lactobacillus	1.4	0.2	0.1	0.71	0.34	0.42	0.48

Values are least-squares means of relative abundance ± standard error of the mean (SEM). Significant difference at *p*-value ≤ 0.05.

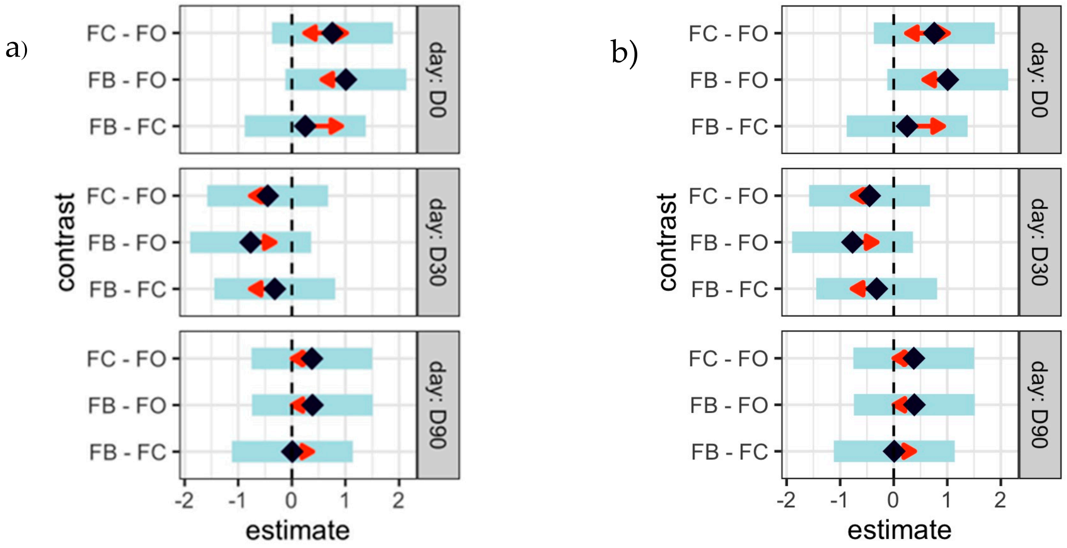

Figure S1 pairwise contrast plots for the three diets (FB, FC, FO) across three timepoints (D0, D30, D90).

a) FDR method (adjust = "fdr"; b) Tukey method (adjust = "tukey")

No statistically significant differences were found. Across all timepoints, pairwise comparisons between the FB, FC, and FO diets revealed no statistically significant differences, as all 95% adjusted confidence intervals overlapped with zero. The most notable trend occurred at Day 0 between the FB and FO diets ($p=0.0888$), but this difference did not reach significance after applying the Tukey adjustment. By Day 90, the mean estimates for all dietary groups converged, showing nearly identical values and high p-values (up to $p=0.9998$) for the comparisons
